# Unraveling the genetic basis of the causal association between inflammatory cytokines and osteonecrosis

**DOI:** 10.3389/fendo.2024.1344917

**Published:** 2024-04-29

**Authors:** Yining Lu, Yan Pei, YiMing Gao, FeiFei Zhao, Ling Wang, Yingze Zhang

**Affiliations:** ^1^ Department of Orthopedic Research Center, the Third Hospital of Hebei Medical University, Shijiazhuang, Hebei, China; ^2^ Department of Orthopedic Surgery, the Third Hospital of Hebei Medical University, Shijiazhuang, Hebei, China; ^3^ Department of Orthopedic Oncology, The Third Hospital of Hebei Medical University, Shijiazhuang, Hebei, China; ^4^ Department of Orthopedic Surgery, the Fourth Hospital of Hebei Medical University, Shijiazhuang, Hebei, China

**Keywords:** bone, necrosis, immune, inflammation, mendelian randomization, GWAS

## Abstract

**Background:**

Previous studies have reported that the occurrence and development of osteonecrosis is closely associated with immune-inflammatory responses. Mendelian randomization was performed to further assess the causal correlation between 41 inflammatory cytokines and osteonecrosis.

**Methods:**

Two-sample Mendelian randomization utilized genetic variants for osteonecrosis from a large genome-wide association study (GWAS) with 606 cases and 209,575 controls of European ancestry. Another analysis included drug-induced osteonecrosis with 101 cases and 218,691 controls of European ancestry. Inflammatory cytokines were sourced from a GWAS abstract involving 8,293 healthy participants. The causal relationship between exposure and outcome was primarily explored using an inverse variance weighting approach. Multiple sensitivity analyses, including MR-Egger, weighted median, simple model, weighted model, and MR-PRESSO, were concurrently applied to bolster the final results.

**Results:**

The results showed that bFGF, IL-2 and IL2-RA were clinically causally associated with the risk of osteonecrosis (OR=1.942, 95% CI=1.13-3.35, p=0.017; OR=0.688, 95% CI=0.50-0.94, p=0.021; OR=1.386, 95% CI=1.04-1.85, p = 0.026). there was a causal relationship between SCF and drug-related osteonecrosis (OR=3.356, 95% CI=1.09-10.30, p=0.034).

**Conclusion:**

This pioneering Mendelian randomization study is the first to explore the causal link between osteonecrosis and 41 inflammatory cytokines. It conclusively establishes a causal association between osteonecrosis and bFGF, IL-2, and IL-2RA. These findings offer valuable insights into osteonecrosis pathogenesis, paving the way for effective clinical management. The study suggests bFGF, IL-2, and IL-2RA as potential therapeutic targets for osteonecrosis treatment.

## Introduction

Osteonecrosis, characterized by bone cell and marrow death, encompasses a group of diseases (1). Triggered by factors like trauma, drugs, alcohol, or blood disorders, it induces an immune response inhibiting bone repair, leading to osteonecrosis (2–6). Although osteonecrosis often occurs in weight-bearing joints, including the hip, knee, and humerus, it can occur in any bone or location, even the craniofacial area (7, 8). It is a progressive disease that imposes a substantial burden on society, especially since it affects young people in their prime working years, and can lead to disability in important weight-bearing joints (9, 10).

The specific etiology and pathophysiology of osteonecrosis remain not fully understood due to its highly heterogeneous nature. Nonetheless, past research underscores the crucial role of the immune inflammatory response in its initiation and progression. Anomalies in immune responses and immune cell infiltration in osteonecrotic tissue often leads to uncontrolled inflammation. While inflammation is integral to bone tissue repair by recruiting immune cells and bone marrow mesenchymal stem cells for assistance (11, 12), persistent inflammation can impede repair and exacerbate failures, particularly in the case of osteonecrosis (6, 13). Nevertheless, we still know very little about the specific inflammatory cytokines involved in osteonecrosis development. Several observational studies in the past have attempted to explore the relationship between inflammatory cytokines and osteonecrosis. Li et al. explored potential inflammation-related biomarkers of osteonecrosis using the Gene Expression Omnibus (GEO) database (14), and Zou et al. explored the relationship between TH17 and IL-17 with osteonecrosis (15). However, the sample sizes of these studies were usually small, resulting in low confidence in the results. In addition, their results may be affected by unforeseen confounding variables or reverse causality because confounding factors were not excluded, which also poses a challenge in establishing a clear causal relationship (16).

Mendelian randomization (MR) analysis methods use genetic variation in nonexperimental data to infer the causal effect of exposure on outcome. Because alleles are randomly assigned during meiosis, MR reduces conventional confounding variables and reverse causality, thus providing better evidence for causal inference (17). Two-sample MR analysis allows researchers to assess instrument-exposure and instrument-outcome associations in two separate population samples, thereby improving the applicability and validity of the test. In this study, we extracted validated genetic variants for 41 inflammatory cytokines for the first time from published genome-wide association study (GWAS) pooled data and innovatively used Mendelian randomization analysis to investigate the association between inflammatory cytokines and osteonecrosis, aiming to fill the gaps in the understanding of the etiology and pathophysiology of osteonecrosis, which utilizes genetic variation as an instrument for the estimation of causality variables, which can alleviate the problem of confounding variables and reverse causality and provide stronger evidence for causal inference.

## Method

### Data resources

**Figure 1:**
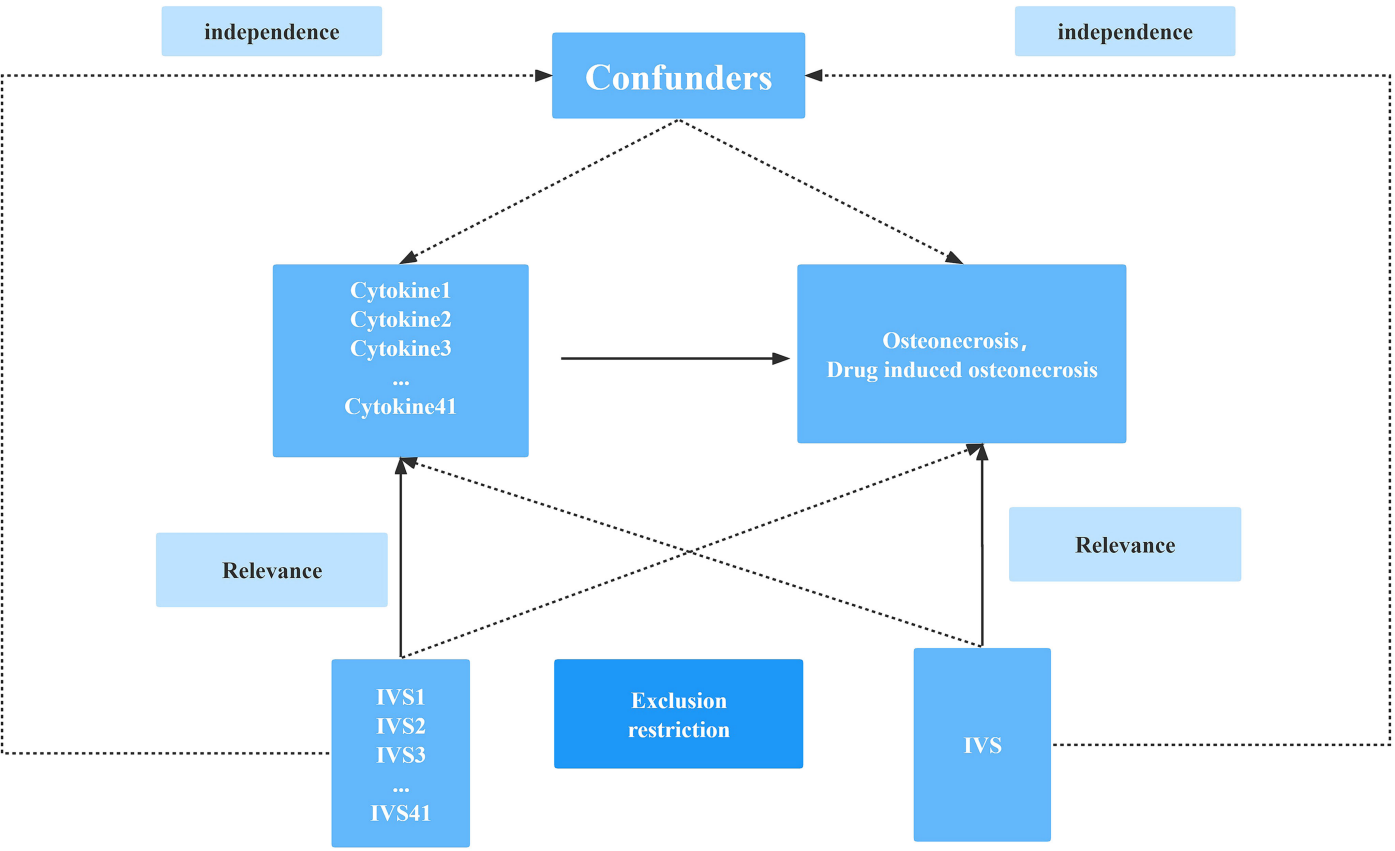
Schematic diagram of the study design in this Mendelian randomization (MR) analysis. Forty-one important instrumental variables for inflammatory cytokines and osteonecrosis were selected and then explored for bidirectional causality. The three basic assumptions of MR analysis, namely correlation, independence, and exclusionary restrictions, are illustrated in this causally directed acyclic graph.

The research design is illustrated in **Figure 1**, incorporating osteonecrosis cases derived from two distinct meta-analysis studies focused on individuals of European ancestry. The first dataset (finn-b-M13_OSTEONECROSIS) comprises 604 cases and 209,575 controls, while the second dataset (finn-b-OSTEON_DRUGS) involves 101 cases and 218,691 controls. Genetic predictors of and genetic associations with cytokines and other systemic inflammatory modulators were obtained from the most recent GWAS of 41 systemic inflammatory modulators in 8293 Finns from three multicenter studies. The three population-based cohorts were: the Cardiovascular Risk in Young Finns Study (mean age of men: 37.4 years; mean age of women: 37.5 years) and the “FINRISK1997” study (mean age of men: 48.3 years; mean age of women: 47.3 years). FINRISK1997” study (mean age of men: 48.3 years; mean age of women: 47.3 years) and “FINRISK2002” study (mean age of men: 60.4 years; mean age of women: 60.1 years). The studies measured circulating inflammatory modulators using Bio-Rad’s premixed Bio-Plex Pro Human Cytokine 27-plex Assay and 21-plex Assay and a Bio-Plex 200 reader with Bio-Plex 6.0 software. Only measurements within the detectable range for each cytokine were considered in the analysis; any cytokine with more than 90% missing values (48 out of 7) was excluded from the analysis. Genetic associations were adjusted for age, sex, body mass index, and the top 10 genetic principal components and corrected for genomic controls. All study participants provided informed consent (18).

### Selection of cytokine SNPs

Mendelian Randomization (MR) analysis relies on three fundamental assumptions: correlation, independence, and exclusion restriction (19). The premise is that the chosen genetic variants exhibit an association with risk factors (correlation) but not with any confounding factors in the risk factor-outcome relationship (independence). Additionally, it assumes that these genetic variants are not linked to the outcome through any pathway other than the targeted risk factor (exclusion restriction). This two-way study integrates data from three Genome-Wide Association Studies (GWAS) encompassing 41 inflammatory cytokines, osteonecrosis, and drug-induced osteonecrosis. The SNP selection process involved applying a genome-wide significance threshold of p < 5 × 10^-8 to identify SNPs strongly associated with osteonecrosis/drug-induced osteonecrosis and inflammatory cytokines. To mitigate linkage disequilibrium, SNPs were clustered with a threshold of kb = 10,000 and r^2 = 0.001. Palindromic SNPs were excluded, considering the challenges in identifying these SNPs in the exposure and outcome GWASs where systemic inflammatory regulators were aligned in the same direction. The R^2 value of each SNP was utilized to compute the proportion of variance in exposure, while the F statistic was employed to estimate instrumental strength, thereby avoiding weak instrumental bias (20, 21). Finally, we will replace the unavailable SNPs in the result summary with the proxy SNPs (r^2 > 0.8) from LDlink (LDlink | An Interactive Web Tool for Exploring Linkage Disequilibrium in Population Groups (nih.gov)) (22).

### Statistical analysis

Given the varying number of SNPs associated with each cytokine, our primary MR analysis for cytokines with only one SNP employed the Wald ratio (23). For those with two or more SNPs, we opted for inverse variance weighting (IVW) as the primary MR analysis (24). This approach aimed to investigate the potential pathogenic role of inflammatory cytokines in relation to the risk of osteonecrosis. To assess the presence of heterogeneity in our primary MR analysis (IVW), we conducted a Cochrane Q test. Most outcomes exhibited no significant heterogeneity (p > 0.05), though a few displayed heterogeneity. It’s important to note that since IVW was our primary analysis and may inherently contain heterogeneity, the presence of heterogeneity in individual outcomes does not significantly impact the overall prediction of causality (25).

To further evaluate causality and explore potential pleiotropy, we implemented additional checks, including MR Egger regression and MR-PRESSO (26). Additionally, a Leave-one-out analysis was performed to examine the influence of individual SNPs on the overall MR analysis. To eliminate potential confounding effects, we utilized PhenoScanner to investigate potential dimorphic phenotypes associated with the evaluated individual SNPs. Much of this analytical work was carried out using R analysis software (version 4.0.3) and relevant R packages, encompassing two-sample MRs, data arrays, and others.

## Result

**Supplementary Table 1** provides comprehensive details about the study and dataset. Notably, all participants in the study were of European descent (100%), a deliberate choice aimed at mitigating the impact of ethnic differences on the research outcomes. This meticulous consideration ensures a homogenous participant pool and enhances the study’s reliability by eliminating potential confounding factors associated with ethnic diversity.

### Selection of instrumental variables

A flowchart outlining the full-text logic is provided in **Figure 1**, and raw data for inflammatory cytokines are available from the link (Cytokines GWAS results - Datasets - data.bris). Genetic variants were screened against screening criteria (P<5×10-8, r^2<0.001, kb=10,000) as described previously. The associations of inflammatory cytokines on osteonecrosis and and drug-induced osteonecrosis are summarized in **Supplementary Tables 2–7**, including chromosomal location, gene, effector allele (EA), other allele and effector allele frequency (EAF). In addition, estimates of the association between each SNP and inflammatory cytokines and osteonecrosis are given, including beta values, standard errors (SE) and P values.

### Causal relationship with osteonecrosis

**Figure 2:**
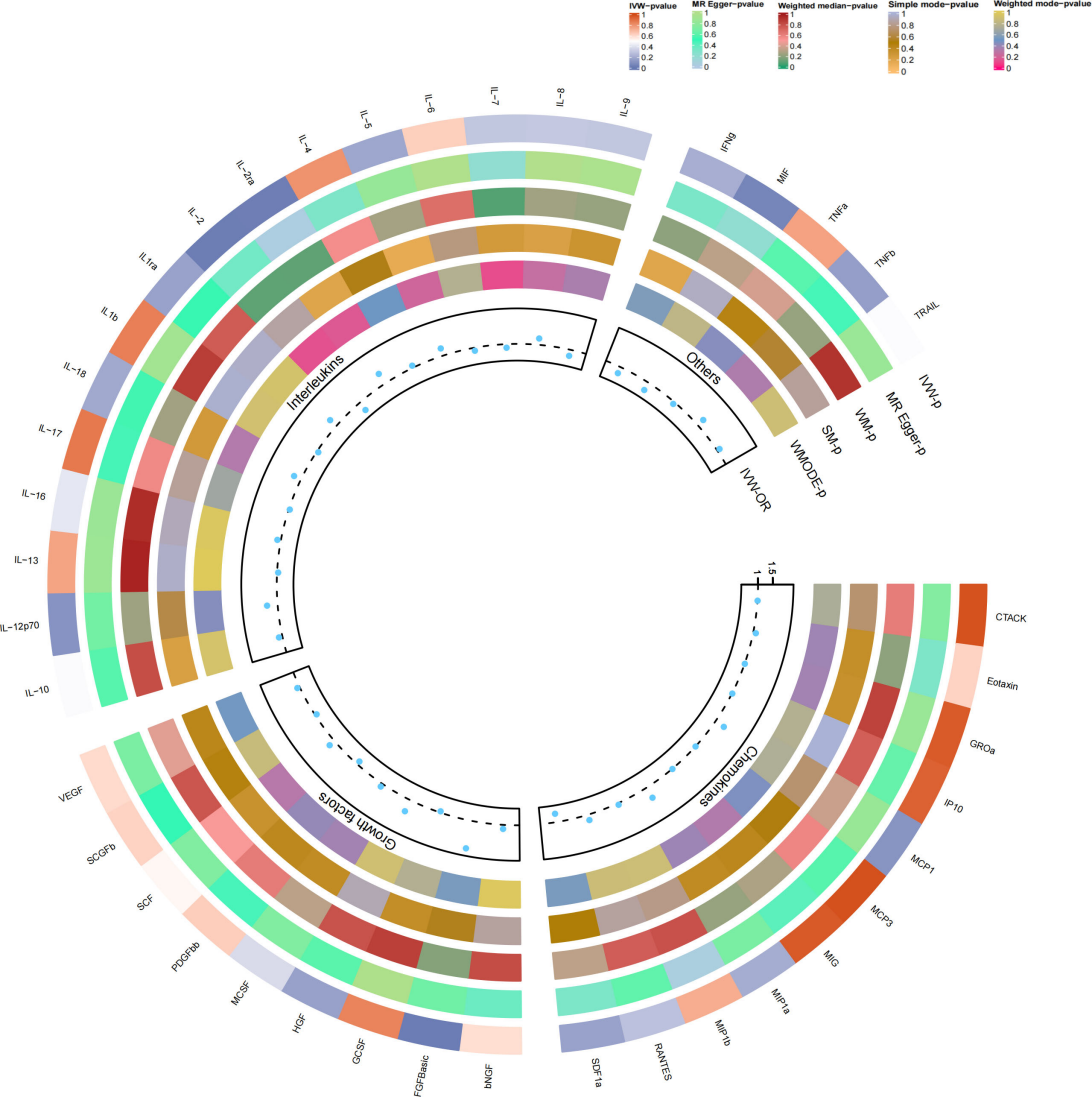
Causal correlations of 41 inflammatory cytokines on osteonecrosis. The change in the odds ratio (OR) of osteonecrosis per one-SD rise in the cytokine level is shown by OR and 95% confidence interval. P-value 0.05/41 = 0.0012 was found significant after multiple-comparison correction. The results from the inverse variance weighted method were shown for all cytokines. bNGF, beta nerve growth factor; CTACK, cutaneous T cell-attracting chemokine; FGFBasic, basic fibroblast growth factor; GCSF, granulocyte colony-stimulating factor; GROa, growth-regulated oncogene-a; HGF, hepatocyte growth factor; IFNg, interferon gamma; IL, interleukin; IP, interferon gamma-induced protein 10; MCP1, monocyte chemotactic protein 1; MCP3, monocyte-specific chemokine 3; MCSF, macrophage colony-stimulating factor; MIF, macrophage migration inhibitory factor; MIG, monokine induced by interferon gamma; MIP1a, macrophage inflammatory protein-1a; MIP1b, macrophage inflammatory protein-1b; PDGFbb, platelet-derived growth factor BB; RANTES, regulated upon activation normal T cell expressed and secreted factor; SCF, stem cell factor; SCGFb, stem cell growth factor beta; SDF1a, stromal cell-derived factor-1 alpha; SNPs, single-nucleotide polymorphisms; TNFa, tumor necrosis factor alpha; TNFb, tumor necrosis factor beta; TRAIL, TNF-related apoptosis-inducing ligand; VEGF, vascular endothelial growth factor.

Our findings suggest a potential involvement of cyclic bFGF, IL-2RA, and IL-2 in the risk of osteonecrosis development, as indicated by the IVW approach (refer to **Figure 2**). Utilizing the IVW method, we observed that elevated genetic prediction levels of cyclic bFGF correlated with an increased risk of osteonecrosis (OR=1.942, 95% CI=1.13-3.35, p=0.017 per 1 standard deviation (SD)) (see **Supplementary Figure 1**). Cochran’s Q test did not reveal any heterogeneity (P=0.858), and directional polymorphisms were absent (MR Egger-intercept=0.038, P=0.717 for MR Egger-intercept; P=0.858 for MR PRESSO global test).

Furthermore, heightened IL-2RA levels were associated with an elevated risk of osteonecrosis (OR=1.386, 95% CI=1.04-1.85, p=0.026), while increased IL-2 levels were linked to a decreased risk (OR=0.688, 95% CI=0.50-0.94, p=0.021, per 1 standard deviation (SD) increase). Cochran’s Q test indicated no heterogeneity (p=0.523; p=0.681), and no directional polymorphism was found (MR Egger-intercept = -0.085, p = 0.135 for MR Egger-intercept, p = 0.568 for MR PRESSO global test; MR Egger-intercept = -0.024, p = 0.689 for MR Egger-intercept, p = 0.736 for MR PRESSO global test) (refer to **Supplementary Tables 2–4**). With the exception of bFGF, IL-2RA, and IL-2, other cytokines such as VEGF, GRO-α, Trail, MIG, IL-7, and IL-17 did not demonstrate any association with osteonecrosis risk in the IVW primary MR analysis or secondary analyses. Notably, our heterogeneity test revealed significant heterogeneity in IL-4 (p=0.015), MIG (p=0.036), and PDGFbb (p=0.042), whereas most other cytokines exhibited significant non-heterogeneity. Aside from MIP1b (p=0.045 for MR Egger-intercept), MR-Egger regression did not identify any polymorphisms in p-values for all cytokines. Our MR-PRESSO assay, serving as an additional robustness test, did not identify any outliers, except for IL-4 (p = 0.01) and MIG (p < 0.04).

### Causal relationship with drug-induced osteonecrosis

**Figure 3:**
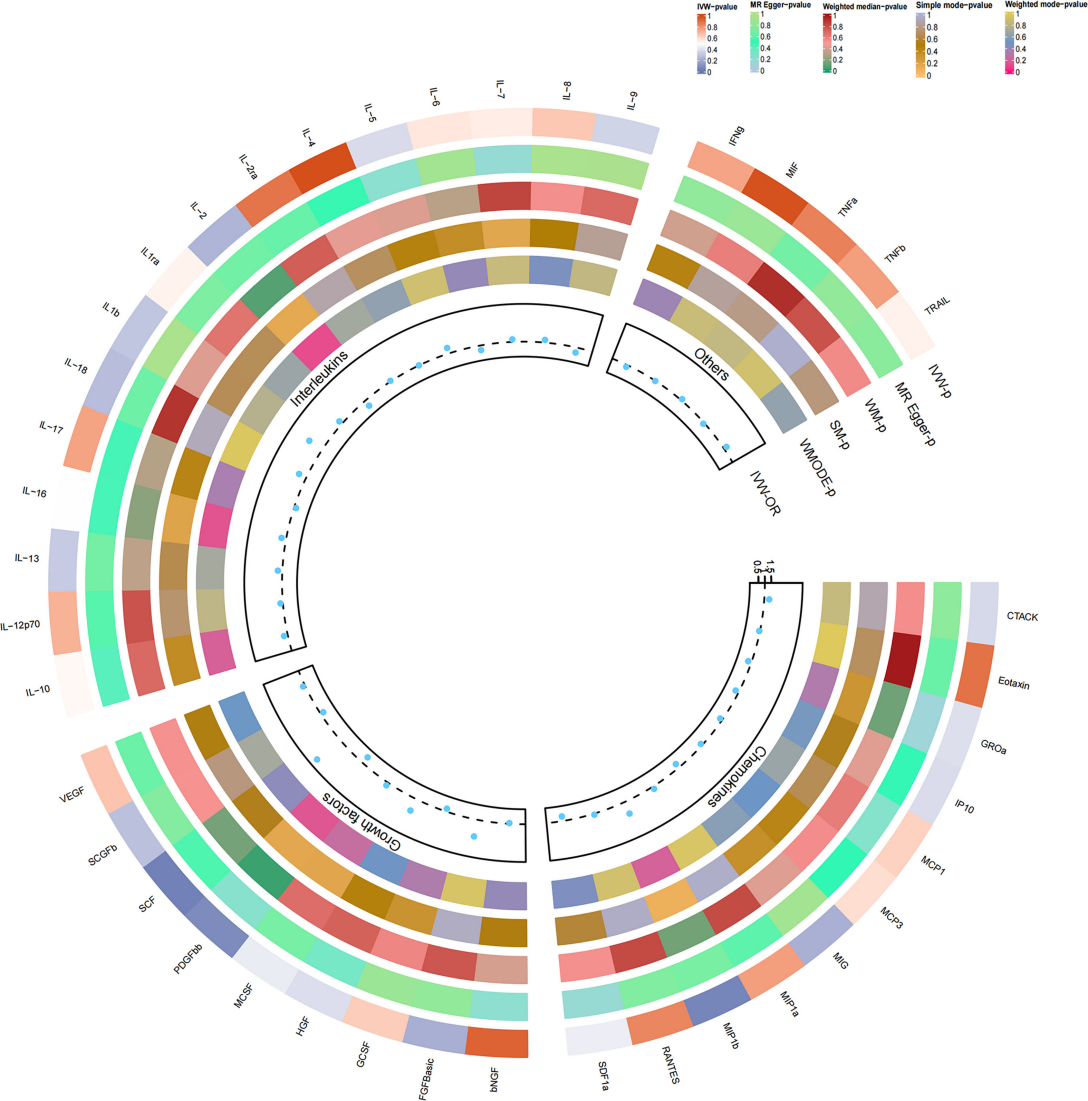
Causal correlations of 41 inflammatory cytokines on drug-induced osteonecrosis. The change in the odds ratio (OR) of drug-induced osteonecrosis per one-SD rise in the cytokine level is shown by OR and 95% confidence interval. P-value 0.05/41 = 0.0012 was found significant after multiple-comparison correction. The results from the inverse variance weighted method were shown for all cytokines.

**Figure 4:**
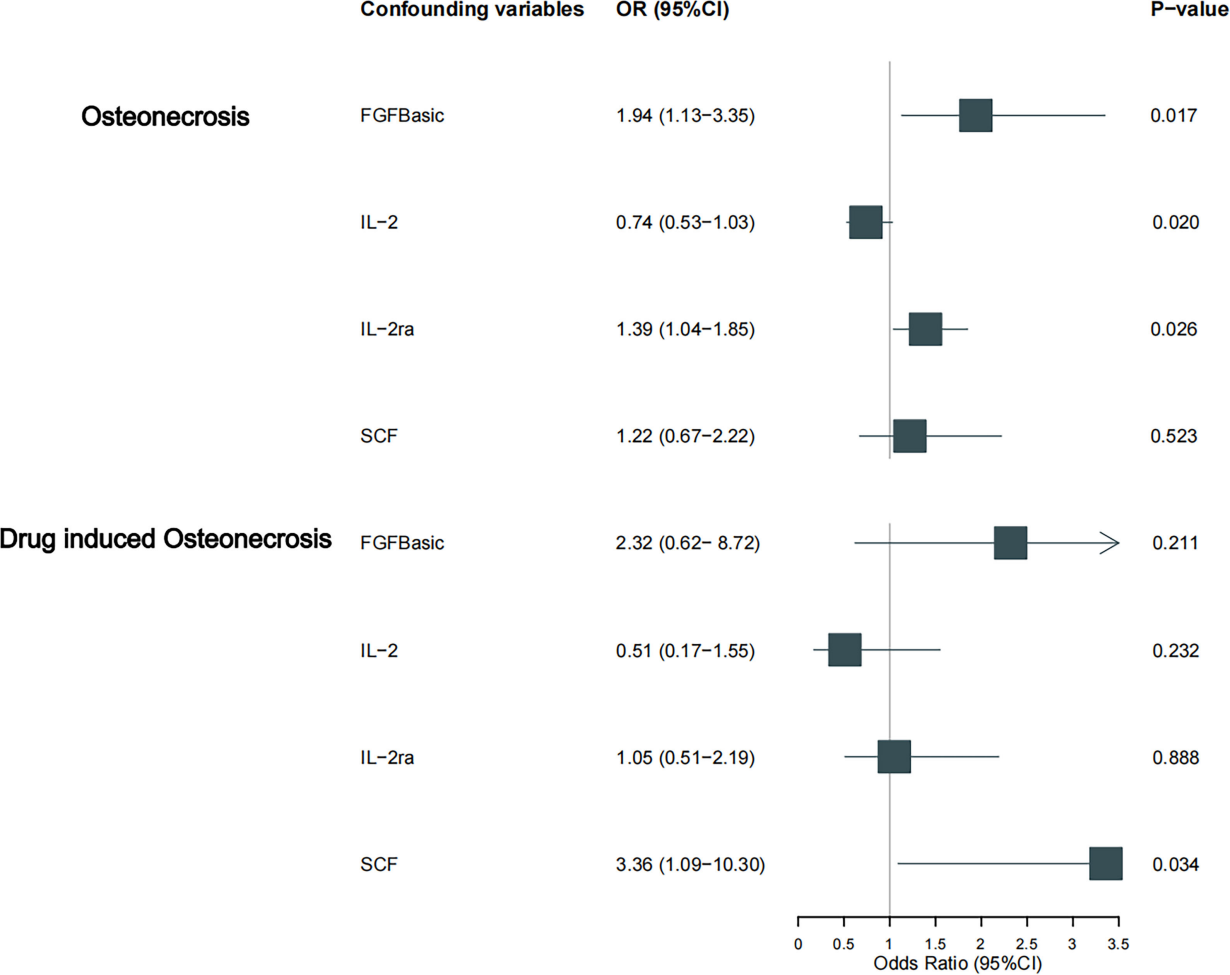
Causal estimation of bFGF, IL-2, IL-2RA and SCF on osteonecrosis and its drug-induced subtype. Forest plots depict the causal estimates of bFGF, IL-2, IL-2RA and SCF on osteonecrosis and its drug-induced subtypes. The dominance ratio (OR) was estimated using the fixed-effects IVW method. Horizontal bars indicate 95% confidence intervals (CI).

As previously mentioned, various factors can contribute to osteonecrosis, with steroid-induced osteonecrosis being a prominent cause of nontraumatic occurrences in nontraumatic fractures. Additionally, osteonecrosis of the jaw induced by bisphosphonates has emerged as a significant area of research interest in recent years. Despite differing mechanisms of action, these drugs share a common outcome—they induce osteonecrosis by disrupting the delicate balance between bone resorption and formation, ultimately interfering with the bone remodeling process. To enhance the robustness of our study, we delved into the causal effects of inflammatory cytokines on the risk of drug-induced osteonecrosis. Notably, bFGF with genetically predicted levels of IL-2RA exhibited a positive, though not statistically significant, causal effect on drug-induced osteonecrosis (OR=2.324, 95% CI=0.62-8.72, p=0.211 per 1 standard deviation (SD); OR=1.053, 95% CI=0.51-2.19, per 1 standard deviation (SD) P=0.888). Similarly, the genetic prediction level of IL-2 demonstrated a negative, albeit not statistically significant, causal effect with drug-induced osteonecrosis (OR=0.507, 95% CI=0.17-1.55, per 1 standard deviation (SD) P=0.232). This trend aligns with the earlier study results, ensuring consistency (**Figures 3**, **4**). Furthermore, in drug-induced osteonecrosis, a higher genetic prediction level of cyclic SCF was associated with an elevated risk of osteonecrosis according to the IVW approach (OR=3.356, 95% CI=1.09-10.30, P=0.034 per 1 standard deviation (SD)) (**Supplementary Figure 2**). Importantly, Cochran’s Q test did not reveal heterogeneity (p=0.667), and directional polymorphisms were absent (MR Egger-intercept=0.071, p=0.655 for MR Egger-intercept; p=0.673 for MR PRESSO global test) (**Supplementary Tables 5–7**).

## Discussion

In our current investigation, our findings strongly indicate a causal relationship between elevated levels of bFGF/IL-2RA and an increased risk of osteonecrosis. Moreover, higher circulating levels of IL-2 appear to be suggestively associated with a reduced risk of osteonecrosis. Additionally, we identified a positive causal effect of SCF on drug-induced osteonecrosis. These results contribute novel evidence to the etiological understanding of osteonecrosis, underscoring the advantages of employing the MR approach. It is noteworthy that our study represents the inaugural application of MR analysis to ascertain whether heightened levels of inflammatory cytokines are correlated with an elevated risk of osteonecrosis. This exploration is based on genetic data extracted from publicly available databases, marking a significant contribution to the field by leveraging robust and unbiased genetic information.

**Figure 5:**
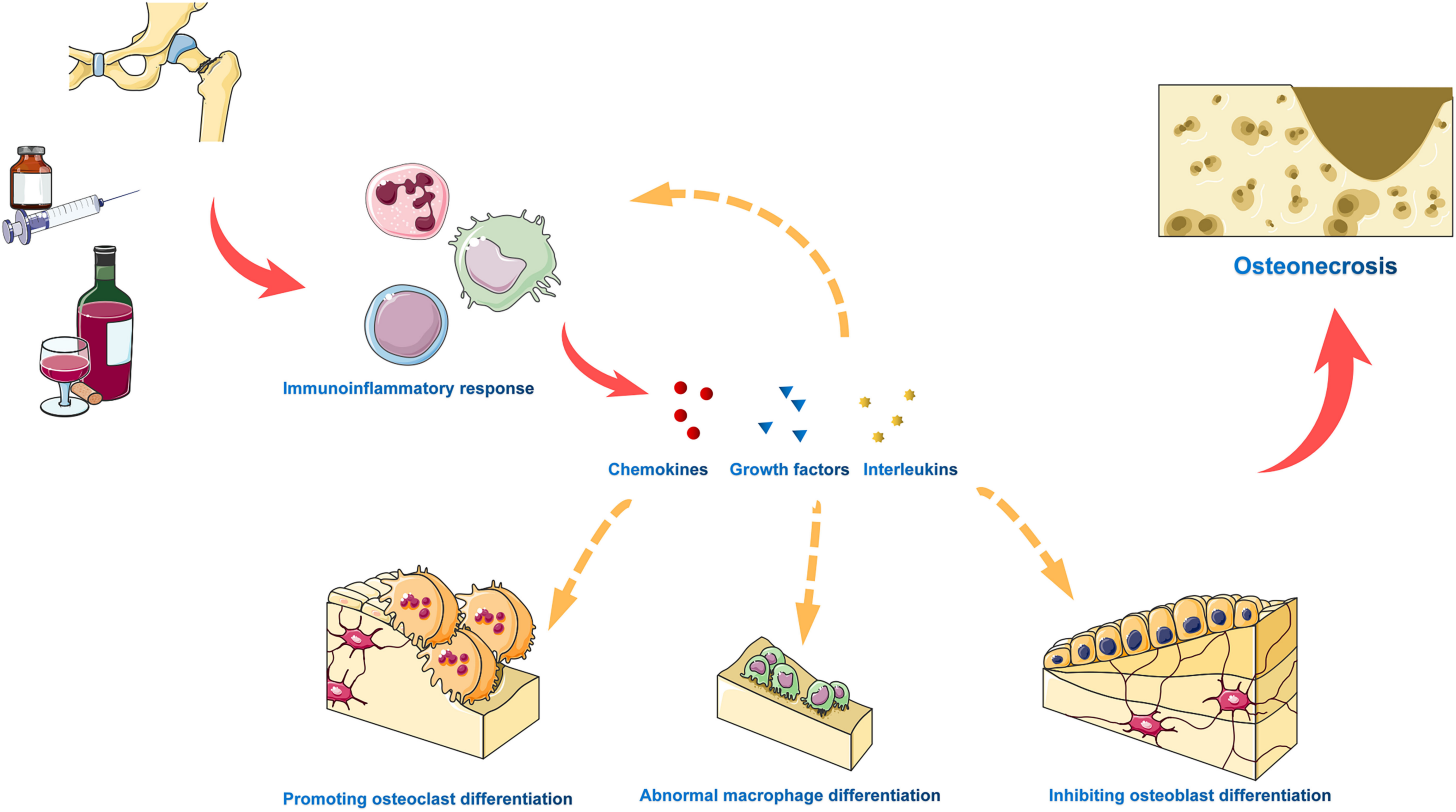
The immune inflammatory response plays a critical role in the development and progression of osteonecrosis. Various adverse factors trigger a chronic immune inflammatory response, resulting in sustained production of pro-inflammatory cytokines, progressive tissue damage, and abnormal tissue remodeling. In necrotic bone tissue, inflammatory cytokines and chemokines attract innate immune cells and adaptive immune cells, which release additional inflammatory cytokines in a positive feedback loop to amplify the overall inflammatory response. Furthermore, chronic inflammation excessively activates bone resorption, inhibits bone formation, and drives osteonecrosis.

The immune-inflammatory response plays a pivotal role in both the development and progression of osteonecrosis, representing a prominent feature of the condition (27, 28). Within the necrotic bone tissue, the sustained production of inflammatory cytokines serves as a constant attractant for both innate immune cells, including macrophages, neutrophils, and dendritic cells, and adaptive immune cells like T and B cells. This creates a positive feedback loop, wherein these immune cells release additional inflammatory cytokines, thereby amplifying the overall inflammatory response (29–31). Furthermore, chronic inflammation not only overstimulates bone resorption but also hinders bone formation, thereby contributing to the pathogenesis of osteonecrosis. This disruption of the normal coordination between pro-inflammatory activation and anti-inflammatory silencing during bone repair forms the pathophysiological basis of osteonecrosis (**Figure 5**).

Numerous studies have delved into the relationship between osteonecrosis and the immune-inflammatory response. However, the causal direction and the extent of association between inflammatory cytokines and the risk of osteonecrosis remain unclear, with observational studies constituting the primary mode of investigation. These studies are susceptible to confounding, reverse causality bias, and measurement errors, which limit their ability to provide causal estimates of exposure-outcome effects and, consequently, hinder their utility in informing prevention and treatment strategies. In contrast, MR analysis employs specific genetic variants that meet instrumental variable assumptions to systematically address causality issues in epidemiological studies, thereby mitigating the potential for inherent bias. Moreover, MR analysis is a more cost-effective and feasible approach compared to randomized controlled trials. According to our present MR analysis, bFGF in conjunction with IL-2RA may contribute to the development of osteonecrosis, while IL-2 exhibits an opposing effect. Additionally, our findings underscore the significance of SCF as a crucial factor in promoting the onset of drug-induced osteonecrosis. These insights gleaned from MR analysis offer a valuable contribution to understanding the complex interplay between inflammatory cytokines and osteonecrosis, offering potential avenues for further research and therapeutic exploration.

IL-2 (interleukin-2) is a regulator of the immune and inflammatory response that participates in the regulation of immune cell activation and proliferation (32). IL-2RA (CD25) is the alpha subunit of the IL-2 receptor and is likewise involved in immune inflammatory responses (33, 34). IL-2 plays a multifaceted regulatory role in the development of bone tissue, activating Treg cells to regulate the activity of innate and adaptive immune system cells and suppress the inflammatory response, which reduces the occurrence of local inflammatory responses, protects blood vessels and bone cells from damage (35–37). IL-2 plays a pivotal role in mitigating osteonecrosis and fostering the healing of bone tissue by impacting various cellular processes. Specifically, it influences osteoblast proliferation and differentiation, promotes osteoclast activity, and inhibits the activity of bone-resorbing cells. Osteoblasts, being crucial for bone repair, are significantly influenced by IL-2, as evidenced by Reyes-Botella et al.’s study, which demonstrated that IL-2 fosters an increase in the number of osteoblasts (38). Furthermore, a vitro research by Sartori et al. suggests that IL-2 can stimulate the upregulation of glucocorticoid receptors, indirectly fostering osteoblast proliferation (39). Additionally, a finding by Sun et al. suggests that IL-2 can hinder osteoclastogenesis, thus indirectly safeguarding bone tissue and aiding in the alleviation of osteonecrosis (40). Due to the limited number of observational clinical studies or meta-analyses linking IL-2RA to osteoimmunity, little is known about the role of IL-2RA in bone tissue healing. IL-2RA has been shown to be associated with the development of autoimmune inflammatory diseases (41, 42), and immune disorders affecting the skeleton may be an important cause of osteonecrosis, with a strong association between abnormal immune responses and immune cell infiltration in osteonecrosis tissue, often resulting in necrotic bone tissue showing signs of uncontrolled inflammation (43, 44). Rheumatoid arthritis (RA) is an autoimmune inflammatory disease that leads to persistent synovitis and severe bone and cartilage destruction, which is closely associated with osteonecrosis (45). Lakshmanan et al. showed that IL-2RA may be clearly linked to the pathogenesis of arthritis (46), and Hinks et al. stated in an earlier study that IL-2RA was juvenile idiopathic arthritis sensitive gene locus, which may reveal another important pathogenesis of osteonecrosis (47). However, the precise mechanisms of IL-2 and IL-2RA in this regard demand further research and exploration to provide a more precise scientific basis and clinical application outlook for the treatment of diseases associated with the skeletal field.

Fibroblast growth factor (FGF) comprises a peptide family with 22 distinct peptides (48), of which basic fibroblast growth factor (bFGF) is a key coordinating factor that affects bone homeostasis. Kawaguchi et al. demonstrated that bFGF, acting on osteoblasts at high concentrations, not only efficiently stimulates bone formation via COX-2 induction and production of prostaglandins, but also stimulates bone resorption, whereas at low concentrations it acts directly on mature osteoclasts to resorb bone (49). In a follow-up study, Kawaguchi demonstrated that bFGF affects bone resorption by acting directly on osteoclasts through activation of FGF receptor 1 and p42/p44 MAP kinase, proving the previous study (50). In addition, a study by Taketomi et al. found that bFGF stimulation induced high levels of Sprouty2 expression, inhibited the expression of markers of osteoblast differentiation, and also inhibited osteoblast matrix mineralization (51). Previous investigations have highlighted bFGF’s capability to induce the differentiation of bone marrow mesenchymal stem cells into lipogenic cells (52, 53). This induction leads to intraosseous fat deposition, triggering structural alterations in bone that impact blood supply and mechanical properties, ultimately culminating in osteonecrosis (54, 55). In addition, the above arguments are supported by Ganguly et al. who showed that myeloid-specific TGF-β signaling, which promotes bFGF expression, is strongly associated with the development of osteolytic bone lesions, and Lee et al.’s study suggests that bFGF plays a key role in the onset and development of RA, which provides support for the above argument (56, 57). Interestingly, however, it has been shown that bFGF in combination with BMP-2 and VEGF promotes osteogenic differentiation of bone marrow MSCs (58). It has also been consequently investigated as a promising agent for promoting bone tissue healing (59, 60), contributing to the development of novel materials to promote bone tissue healing. As a versatile factor, bFGF is anticipated to emerge as a new therapeutic target for preventing osteonecrosis and promoting bone tissue healing. This multifaceted role positions bFGF as an active participant in the development and prognosis of osteonecrosis, presenting potential avenues for therapeutic interventions.

Our study found an interesting association between stem cell factor (SCF) and a higher risk of developing pharmacological osteonecrosis (OR=3.356, 95% CI=1.09-10.30, p=0.034 per 1 standard deviation (SD)). Earlier studies have demonstrated the potent enhancing effect of SCF on osteoclast activity *in vitro* (61), and glucocorticoids, one of the causative agents of pharmacologic osteonecrosis, also increase the expression of SCF in various ways (62), which may reveal one of the key pathogenetic mechanisms of drug-induced osteonecrosis. Furthermore, it is worth noting that Molfetta et al. demonstrated that sustained stimulation of SCF promotes the proliferation of connective tissue-like mast cell subpopulations and maintains a pro-inflammatory microenvironment in the body, which may also be one of the potential factors contributing to the development of osteonecrosis lesions (63). Interestingly, however, the positive effects of SCF on osteogenesis, possibly through the c-Kit-Akt signaling pathway, have been recognized in recent years, which may also suggest that SCF may have beneficial effects on osteonecrosis repair (64, 65). Additionally, studies have shown that SCF can stimulate cell migration and proliferation, promote blood vessel formation and maturation, and play an important role in angiogenesis and tissue engineering, which may be a potential therapeutic target for osteonecrosis (66, 67). While only a limited number of clinical observational studies or meta-analyses have investigated the correlation between SCF and osteonecrosis or drug-induced osteonecrosis, our current MR analysis presents compelling evidence suggesting that elevated levels of circulating SCF are indeed associated with an increased risk of osteonecrosis. This insight, derived from the unique perspective of MR analysis, contributes valuable information to the existing body of research and emphasizes the importance of considering SCF as a potential factor in the development of osteonecrosis.

The current study has successfully established a causal relationship between bFGF, IL-2, IL-RA, and osteonecrosis from a genetic perspective. Although SCF did not exhibit any statistically significant causal association with osteonecrosis, it remains a potential key player in the mechanism of drug-induced osteonecrosis development. This study holds several notable advantages. Firstly, it is the inaugural MR study to illuminate the relationship between inflammatory cytokines and the risk of osteonecrosis. Secondly, in contrast to observational studies, our approach substantially mitigates the impact of confounders and reverse causality, enhancing the reliability of causality inferences. Thirdly, leveraging publicly available GWAS databases and a substantial amount of original study data contributes to the robustness of our findings. Fourthly, compared to the time-consuming nature of randomized controlled trials (RCTs), our study provides cost-effective insights. Nevertheless, the study does have certain limitations. Firstly, owing to the European origin of the database, the results may not be fully generalizable to other populations. Secondly, the study faced constraints due to the limited dataset of cases and non-cases with osteonecrosis. Given that genetic variation typically exerts a modest effect on exposure, necessitating large sample sizes for statistically significant results, our dataset size might be limiting. In addition, this study only does not have validation against other databases, which may have some impact on the usability of our findings. Lastly, the publicly available pooled meta-analyses lacked comprehensive information on sex and age, and therefore did not allow for subgroup analyses based on demographic or clinical factors, nor did they allow for a comprehensive assessment of potential nonlinear associations between circulating levels of inflammatory factors and osteonecrosis, which may have led to less than comprehensive study results.

## Conclusion

In conclusion, this Mendelian randomization study stands as the inaugural investigation into the causal relationship between osteonecrosis and 41 distinct inflammatory cytokines. Notably, the study unequivocally establishes a causal association between bFGF, IL-2, and IL-2RA with osteonecrosis. These findings not only contribute significant insights into the pathogenesis of osteonecrosis but also pave the way for the development of effective management strategies in clinical settings. The identification of bFGF, IL-2, and IL-2RA as causally linked to osteonecrosis suggests promising avenues for potential therapeutic interventions. This novel perspective positions these cytokines as potential targets for the treatment of osteonecrosis, offering hope for the development of more targeted and effective therapeutic approaches in the future. However, it’s noted that due to the European origin of the database, the generalizability of the findings to other populations, beyond individuals of European ancestry, may be limited.

## Data availability statement

The original contributions presented in the study are included in the article/**Supplementary Material**. Further inquiries can be directed to the corresponding authors.

## Author contributions

YL: Conceptualization, Writing – original draft. YP: Conceptualization, Writing – original draft, Data curation. Y-MG: Investigation, Methodology, Writing – original draft. F-FZ: Writing – review & editing. LW: Writing – review & editing, Conceptualization. YZ: Conceptualization, Writing – review & editing.
