# Supplementary material for: Unraveling the genetic basis of the causal association between inflammatory cytokines and osteonecrosis
Source: Front Endocrinol (Lausanne). 2024 Apr 29;15:1344917. doi: 10.3389/fendo.2024.1344917 (PMC11091469; doi:10.3389/fendo.2024.1344917)
Supplement: Supplementary file 8 [file DataSheet_1.docx]

**STROBE-MR checklist of recommended items to address in reports of Mendelian randomization studies**^1^ ^2^

| **Item No.** | **Section** | **Checklist item** | **Page No.** | **Relevant text from manuscript** |
| --- | --- | --- | --- | --- |
| 1 | **TITLE and ABSTRACT** | Indicate Mendelian randomization (MR) as the study’s design in the title and/or the abstract if that is a main purpose of the study | 1 | Genetic perspectives on the influence of circulating cytokines on acne: A Mendelian randomization studyy |
|  | **INTRODUCTION** |  |  |  |
| 2 | **Background** | Explain the scientific background and rationale for the reported study. What is the exposure? Is a potential causal relationship between exposure and outcome plausible? Justify why MR is a helpful method to address the study question | 2 | Acne is a common skin condition that primarily affects adolescents....large cohort genome-wide association studies |
| 3 | **Objectives** | State specific objectives clearly, including pre-specified causal hypotheses (if any). State that MR is a method that, under specific assumptions, intends to estimate causal effects | 3 | Acne is a common skin condition that primarily affects adolescents....large cohort genome-wide association studies |
|  | **METHODS** |  |  |  |
| 4 | **Study design and data sources** | Present key elements of the study design early in the article. Consider including a table listing sources of data for all phases of the study. For each data source contributing to the analysis, describe the following: |  |  |
|  | a) | Setting: Describe the study design and the underlying population, if possible. Describe the setting, locations, and relevant dates, including periods of recruitment, exposure, follow-up, and data collection, when available. | 3 | The study design is outlined in Figure 1. ...All participants provided written informed consent. |
|  | b) | Participants: Give the eligibility criteria, and the sources and methods of selection of participants. Report the sample size, and whether any power or sample size calculations were carried out prior to the main analysis | 3 | The study design is outlined in Figure 1. ...All participants provided written informed consent. |
|  | c) | Describe measurement, quality control and selection of genetic variants | 4 | MR analysis has three core assumptions, namely correlation, independence, and exclusion restriction...from LDlink (LDlink \| An Interactive Web Tool for Exploring Linkage Disequilibrium in Population Groups (nih.gov)) |
|  | d) | For each exposure, outcome, and other relevant variables, describe methods of assessment and diagnostic criteria for diseases | 4 | MR analysis has three core assumptions, namely correlation, independence, and exclusion restriction...from LDlink (LDlink \| An Interactive Web Tool for Exploring Linkage Disequilibrium in Population Groups (nih.gov)) |
|  | e) | Provide details of ethics committee approval and participant informed consent, if relevant | 4 | Not applicable |
| 5 | **Assumptions** | Explicitly state the three core IV assumptions for the main analysis (relevance, independence and exclusion restriction) as well assumptions for any additional or sensitivity analysis | 4 | MR analysis has three core assumptions, namely correlation, independence, and exclusion restriction...from LDlink (LDlink \| An Interactive Web Tool for Exploring Linkage Disequilibrium in Population Groups (nih.gov)) |
| 6 | **Statistical methods: main analysis** | Describe statistical methods and statistics used |  |  |
|  | a) | Describe how quantitative variables were handled in the analyses (i.e., scale, units, model) | 4 | It is assumed that the selected genetic variants are associated with risk factors (correlation) but not with any confounders in the risk factor-outcome association (independence) and that they are not associated with the outcome through any pathway other than the risk factor of interest (exclusion restriction). |
|  | b) | Describe how genetic variants were handled in the analyses and, if applicable, how their weights were selected | 4 | First, we used p < 5 × 10-8 as a genome-wide significance threshold...rom LDlink (LDlink \| An Interactive Web Tool for Exploring Linkage Disequilibrium in Population Groups (nih.gov)) |
|  | c) | Describe the MR estimator (e.g. two-stage least squares, Wald ratio) and related statistics. Detail the included covariates and, in case of two-sample MR, whether the same covariate set was used for adjustment in the two samples | 4 | It is assumed that the selected genetic variants are associated with risk factors (correlation) but not with any confounders in the risk factor-outcome association (independence) and that they are not associated with the outcome through any pathway other than the risk factor of interest (exclusion restriction). |
|  | d) | Explain how missing data were addressed | 4 | If the SNP as IV contain missing data in the exposure or outcome  summary, it would be omitted |
|  | e) | If applicable, indicate how multiple testing was addressed | 4 | Since each cytokine has a different number of SNP...including two sample MRs, data arrays, etc. |
| 7 | **Assessment of assumptions** | Describe any methods or prior knowledge used to assess the assumptions or justify their validity | 4 | Since each cytokine has a different number of SNP...including two sample MRs, data arrays, etc. |
| 8 | **Sensitivity analyses and additional analyses** | Describe any sensitivity analyses or additional analyses performed (e.g. comparison of effect estimates from different approaches, independent replication, bias analytic techniques, validation of instruments, simulations) | 4 | Since each cytokine has a different number of SNP...including two sample MRs, data arrays, etc. |
| 9 | **Software and pre-registration** |  |  |  |
|  | a) | Name statistical software and package(s), including version and settings used | 4 | TwoSample MR package and MR-PRESSO in R (version 4.0.3) were used to conduct the analysis. |
|  | b) | State whether the study protocol and details were pre-registered (as well as when and where) | 4 | TwoSample(22) MR package and MR-PRESSO(19) in R (version  4.1.2) were used to conduct the analysis. |
|  | **RESULTS** |  |  |  |
| 10 | **Descriptive data** |  |  |  |
|  | a) | Report the numbers of individuals at each stage of included studies and reasons for exclusion. Consider use of a flow diagram | 5 | A flowchart outlining the full text logic is provided in Figure 1...except for EOTAXIN (p = 0.01) and GROa (p < 0.01). |
|  | b) | Report summary statistics for phenotypic exposure(s), outcome(s), and other relevant variables (e.g. means, SDs, proportions) | 5 | A flowchart outlining the full text logic is provided in Figure 1...except for EOTAXIN (p = 0.01) and GROa (p < 0.01). |
|  | c) | If the data sources include meta-analyses of previous studies, provide the assessments of heterogeneity across these studies | 5 | A flowchart outlining the full text logic is provided in Figure 1...except for EOTAXIN (p = 0.01) and GROa (p < 0.01). |
|  | d) | For two-sample MR:  i.  Provide justification of the similarity of the genetic variant-exposure associations between the exposure and outcome samples  ii.  Provide information on the number of individuals who overlap between the exposure and outcome studies | 5 | A flowchart outlining the full text logic is provided in Figure 1...except for EOTAXIN (p = 0.01) and GROa (p < 0.01). |
| 11 | **Main results** |  |  |  |
|  | a) | Report the associations between genetic variant and exposure, and between genetic variant and outcome, preferably on an interpretable scale | 5 | A flowchart outlining the full text logic is provided in Figure 1...except for EOTAXIN (p = 0.01) and GROa (p < 0.01). |
|  | b) | Report MR estimates of the relationship between exposure and outcome, and the measures of uncertainty from the MR analysis, on an interpretable scale, such as odds ratio or relative risk per SD difference | 5 | A flowchart outlining the full text logic is provided in Figure 1...except for EOTAXIN (p = 0.01) and GROa (p < 0.01). |
|  | c) | If relevant, consider translating estimates of relative risk into absolute risk for a meaningful time period | Not  available | Not provided in original research |
|  | d) | Consider plots to visualize results (e.g. forest plot, scatterplot of associations between genetic variants and outcome versus between genetic variants and exposure) | 5 | A flowchart outlining the full text logic is provided in Figure 1...except for EOTAXIN (p = 0.01) and GROa (p < 0.01). |
| 12 | **Assessment of assumptions** |  |  |  |
|  | a) | Report the assessment of the validity of the assumptions | 5 | A flowchart outlining the full text logic is provided in Figure 1...except for EOTAXIN (p = 0.01) and GROa (p < 0.01). |
|  | b) | Report any additional statistics (e.g., assessments of heterogeneity across genetic variants, such as *I^2^*, Q statistic or E-value) | 5 | A flowchart outlining the full text logic is provided in Figure 1...except for EOTAXIN (p = 0.01) and GROa (p < 0.01). |
| 13 | **Sensitivity analyses and additional analyses** |  |  |  |
|  | a) | Report any sensitivity analyses to assess the robustness of the main results to violations of the assumptions | 5 | A flowchart outlining the full text logic is provided in Figure 1...except for EOTAXIN (p = 0.01) and GROa (p < 0.01). |
|  | b) | Report results from other sensitivity analyses or additional analyses | 5 | A flowchart outlining the full text logic is provided in Figure 1...except for EOTAXIN (p = 0.01) and GROa (p < 0.01). |
|  | c) | Report any assessment of direction of causal relationship (e.g., bidirectional MR) | 5 | A flowchart outlining the full text logic is provided in Figure 1...except for EOTAXIN (p = 0.01) and GROa (p < 0.01). |
|  | d) | When relevant, report and compare with estimates from non-MR analyses | 5 | A flowchart outlining the full text logic is provided in Figure 1...except for EOTAXIN (p = 0.01) and GROa (p < 0.01). |
|  | e) | Consider additional plots to visualize results (e.g., leave-one-out analyses) | 5 | A flowchart outlining the full text logic is provided in Figure 1...except for EOTAXIN (p = 0.01) and GROa (p < 0.01). |
|  | **DISCUSSION** |  |  |  |
| 14 | **Key results** | Summarize key results with reference to study objectives | 5 | Acne is a common skin condition that is caused by a combination of factors including genetics...and MR does not address the dynamics of cytokine levels. |
| 15 | **Limitations** | Discuss limitations of the study, taking into account the validity of the IV assumptions, other sources of potential bias, and imprecision. Discuss both direction and magnitude of any potential bias and any efforts to address them | 5 | Acne is a common skin condition that is caused by a combination of factors including genetics...and MR does not address the dynamics of cytokine levels. |
| 16 | **Interpretation** |  |  |  |
|  | a) | Meaning: Give a cautious overall interpretation of results in the context of their limitations and in comparison with other studies | 10 | Acne is a common skin condition that is caused by a combination of factors including genetics...and MR does not address the dynamics of cytokine levels. |
|  | b) | Mechanism: Discuss underlying biological mechanisms that could drive a potential causal relationship between the investigated exposure and the outcome, and whether the gene-environment equivalence assumption is reasonable. Use causal language carefully, clarifying that IV estimates may provide causal effects only under certain assumptions | 11-12 | Acne is a common skin condition that is caused by a combination of factors including genetics...and MR does not address the dynamics of cytokine levels. |
|  | c) | Clinical relevance: Discuss whether the results have clinical or public policy relevance, and to what extent they inform effect sizes of possible interventions | 11-12 | Acne is a common skin condition that is caused by a combination of factors including genetics...and MR does not address the dynamics of cytokine levels. |
| 17 | **Generalizability** | Discuss the generalizability of the study results (a) to other populations, (b) across other exposure periods/timings, and (c) across other levels of exposure | 11-12 | Acne is a common skin condition that is caused by a combination of factors including genetics...and MR does not address the dynamics of cytokine levels. |
|  | **OTHER INFORMATION** |  |  |  |
| 18 | **Funding** | Describe sources of funding and the role of funders in the present study and, if applicable, sources of funding for the databases and original study or studies on which the present study is based | 15 | Acne is a common skin condition that is caused by a combination of factors including genetics...and MR does not address the dynamics of cytokine levels. |
| 19 | **Data and data sharing** | Provide the data used to perform all analyses or report where and how the data can be accessed, and reference these sources in the article. Provide the statistical code needed to reproduce the results in the article, or report whether the code is publicly accessible and if so, where | 15 | Acne is a common skin condition that is caused by a combination of factors including genetics...and MR does not address the dynamics of cytokine levels. |
| 20 | **Conflicts of Interest** | All authors should declare all potential conflicts of interest | 15 | Acne is a common skin condition that is caused by a combination of factors including genetics...and MR does not address the dynamics of cytokine levels. |
