# Supplementary material for: Unraveling the genetic basis of the causal association between inflammatory cytokines and osteonecrosis
Source: Front Endocrinol (Lausanne). 2024 Apr 29;15:1344917. doi: 10.3389/fendo.2024.1344917 (PMC11091469; doi:10.3389/fendo.2024.1344917)
Supplement: Supplementary file 10 [file DataSheet_3.docx]

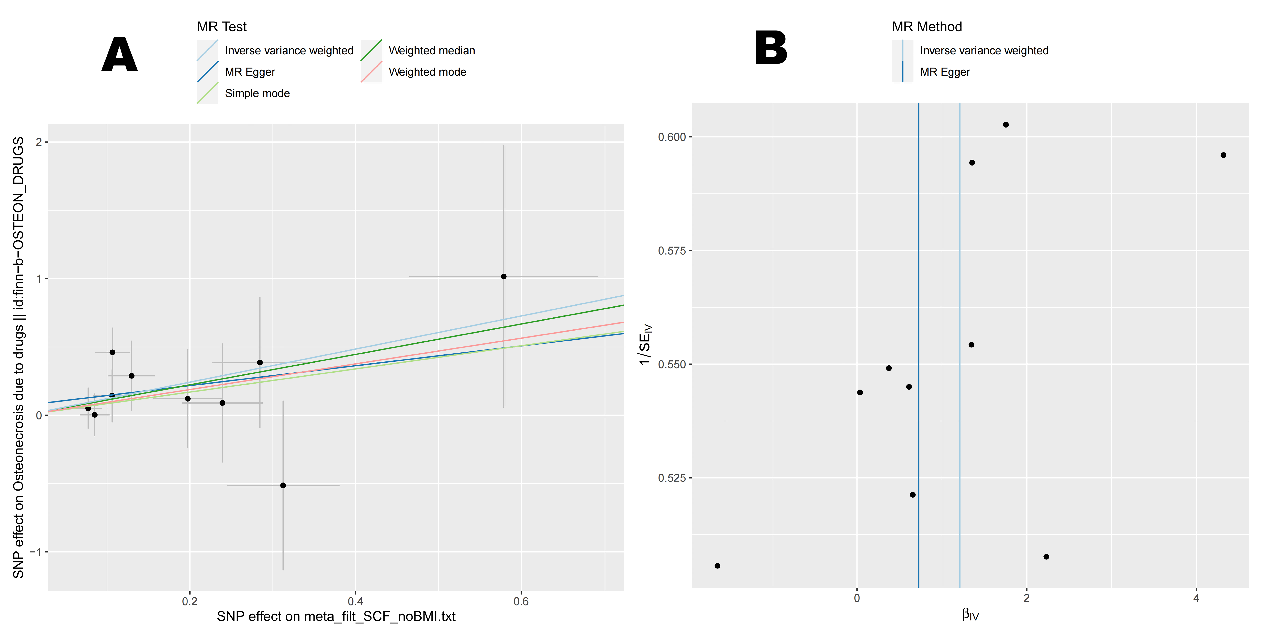


**Supplementary Figure 2**. Scatter plots and funnel plots of Mendelian randomization (MR) analyses for SCF in drug-induced osteonecrosis. (A) Individual inverse variance (IV) associations with cytokine risk are displayed versus individual IV associations with osteonecrosis in black dots. The 95%CI of odd ratio for each IV is shown by vertical and horizontal lines. The slope of the lines represents the estimated causal effect of the MR methods. (B) The funnel plots show the inverse variance weighted MR estimate of each cytokine single-nucleotide polymorphism with osteonecrosis versus 1/standard error (1/SEIV).
